# Negative Weight Attitudes and Disordered Eating Behaviors in Hispanic Adolescents: A Descriptive Study of Gender and Weight Status Associations

**DOI:** 10.3390/jcm14155211

**Published:** 2025-07-23

**Authors:** Tabbetha D. Lopez, Aliye B. Cepni, Katherine R. Hendel, Lenora P. Goodman, Margit Wiesner, Craig A. Johnston, Kevin Haubrick, Tracey A. Ledoux

**Affiliations:** 1 Department of Human Sciences, Sam Houston State University, Huntsville, TX 77341, USA; 2 Department of Health and Human Performance, University of Houston, Houston, TX 77204, USA; abcepni@cougarnet.uh.edu (A.B.C.); cajohn25@central.uh.edu (C.A.J.); khaubric@central.uh.edu (K.H.); taledoux@central.uh.edu (T.A.L.); 3 Division of Epidemiology and Community Health, University of Minnesota School of Public Health, Minneapolis, MN 55455, USA; karlingh@umn.edu (K.R.H.); goodm315@umn.edu (L.P.G.); 4 Department of Psychological, Health, and Learning Sciences, University of Houston, Houston, TX 77204, USA; mwiesner@central.uh.edu

**Keywords:** eating disorder, Modified Kids Eating Disorder Survey, prevention, body dissatisfaction, weight

## Abstract

**Background/Objectives**: Hispanic adolescents experience elevated rates of disordered eating behaviors and body dissatisfaction, yet limited research has examined how gender and weight status interact to shape these risks within this population. **Methods**: A cross-sectional survey was conducted among 680 Hispanic adolescents (ages 9–15) from a predominantly Mexican-American middle school. Participants completed the Modified Kids Eating Disorder Survey (M-KEDS), and height and weight were objectively measured to determine BMI-for-age percentile. Chi-square tests, Mann–Whitney U tests, and logistic regression were used to assess differences by gender and weight status, including interaction effects. Bonferroni correction was applied for multiple comparisons. Effect sizes (Cramér’s V, odds ratios with 95% CI) were reported. **Results**: Approximately 73% of participants reported body dissatisfaction, with significant differences observed by gender and weight status. Adolescents with overweight/obesity reported significantly higher negative weight attitudes and extreme weight control behaviors than healthy-weight peers (*p* < 0.001), with large effect sizes. Females endorsed more disordered attitudes and behaviors, except for exercise to lose weight, which was more common among overweight/obese males. **Conclusions**: These findings underscore the high prevalence and significance of disordered eating behaviors in Hispanic adolescents, including those at a healthy weight. Results highlight the importance of culturally tailored, gender-sensitive screening and prevention strategies. Schools serve as critical settings for early identification, and tools like the M-KEDS can help address disparities in care access and improve outcomes among Hispanic youth.

## 1. Introduction

An eating disorder diagnosis, as defined in the *Diagnostic and Statistical Manual of Mental Disorders, Fifth Edition* (DSM-5), is characterized by severe and frequent disordered eating behaviors, excessive exercise, and an unhealthy preoccupation with one’s weight or shape [1]. Eating disorders take many different forms. Anorexia nervosa (AN), bulimia nervosa (BN), and binge eating disorder (BED) are the most common [1]. The health consequences of eating disorders include muscle wasting, heart failure, cardiovascular complications, gastroparesis, constipation, pancreatitis, bone loss, and kidney failure [2]. Approximately half of adults with eating disorder diagnoses report serious interference in daily activities, relationships, and their ability to fulfill responsibilities [3]. Among adolescents, about 3% have a diagnosed eating disorder, with Hispanic adolescents at a higher risk for binge eating disorder compared to other racial and ethnic groups [4]. Given the severe mental and physical health consequences of diagnosed eating disorders and the heightened prevalence among the largest minority group in the US, research in this area may have significant health implications for a large portion of the US population.

Disordered eating behaviors, such as extreme dieting, purging, restricting, and bingeing, are precursors to eating disorder diagnoses and can lead to various mental and physical health problems, including depression, anxiety, reduced quality of life, hyperlipidemia, and obesity [5,6,7,8,9]. These behaviors exist on a continuum, ranging from unhealthy (i.e., frequent dieting, excessive exercise) to extreme (i.e., self-induced vomiting, loss of control around food) [10]. Such behaviors are increasingly common among youth [11]. Approximately 42% of all adolescents report engaging in at least one disordered eating behavior [12]. Evidence suggests that Hispanic youth have the highest prevalence of frequent dieting, restriction, and binge eating behaviors compared to other racial and ethnic groups [12,13,14].

Additionally, 11% of Hispanic females and 3% of Hispanic males have reported experiencing at least one extreme disordered eating behavior in the last year, indicating potential gender differences in these behaviors [12,15]. Furthermore, Hispanic males and females with overweight or obesity report significantly greater dieting practices, unhealthy weight control behaviors, overeating, and body dissatisfaction compared to their peers with healthy weight status [10,16,17,18]. This preliminary research highlights potential gender and weight status differences in disordered eating behaviors among Hispanic youth, but further research is needed to confirm these findings.

Body dissatisfaction is a precursor to disordered eating behaviors and diagnosed eating disorders [18,19,20]. Body dissatisfaction refers to a negative assessment of one’s shape or weight [21]. Adolescents with high levels of body dissatisfaction are at greater risk for disordered eating behaviors, low self-esteem, and depressive symptoms [19,20]. Body dissatisfaction is highly prevalent among Hispanic youth, particularly females [22,23]. While some studies suggest males suffer from body dissatisfaction [24], none of these studies have estimated body dissatisfaction rates among Hispanic males specifically. In the overall population, youth with overweight or obesity are more dissatisfied with their bodies relative to those in a healthy weight classification [25,26]. Given that Hispanic youth have higher rates of obesity than other racial and ethnic groups, a large proportion of Hispanic youth may be vulnerable to body dissatisfaction and disordered eating behaviors [27]. Understanding the gender and weight status differences in body dissatisfaction among Hispanic youth may have clinical implications for eating disorder treatment and prevention initiatives.

Prior research has examined gender and weight status independently in relation to disordered eating behaviors. Still, fewer studies have explored how these factors interact, particularly within Hispanic adolescent populations, who may experience unique sociocultural pressures. The current study builds on previous work by investigating how negative weight attitudes and extreme weight control behaviors differ across gender-by-weight status subgroups. This intersectional approach offers a more nuanced understanding of risk patterns and may improve early identification strategies and culturally responsive interventions. The purpose of this study was to examine how gender and weight status predict body dissatisfaction and disordered eating behaviors in Hispanic adolescents using the Modified Kids Eating Disorder Survey (M-KEDS). We hypothesized that adolescents with overweight/obesity would report significantly higher disordered eating behaviors and weight-related attitudes than those with a healthy weight and that gender differences would be more pronounced among adolescents with overweight/obesity.

## 2. Methods

### 2.1. Sample

This study utilizes secondary baseline data from a school-based, intensive lifestyle intervention among Hispanic middle school students at a charter school in Houston, TX (*n* = 760) [28,29,30]. For this study, only participants with complete Modified Kids Eating Disorder Survey (M-KEDS) [31] data and gender information at baseline were included (*n* = 680). One-way univariate analyses of variance tests revealed no significant differences (*p* > 0.05) in demographic characteristics, including age, body mass index (BMI), and body mass index z-score (zBMI), between excluded individuals and those included in the analytical sample. The Baylor College of Medicine’s Institutional Review Board approved the original intervention study. All parents provided written consent, and adolescents provided written assent prior to enrollment in the study.

### 2.2. Measures

#### 2.2.1. Modified Kids Eating Disorder Survey (M-KEDS)

The M-KEDS is a brief, seven-item self-report screening tool designed to assess negative weight attitudes and extreme weight control behaviors in Hispanic adolescents [31]. The negative weight attitude subscale includes four items, three of which are scored as ‘yes’ = 1 and ‘no’ = 0: wanting to lose weight, feeling that one looks fat to others, and fearing weight gain. The final item evaluates body dissatisfaction and involves eight child figure drawings (i.e., silhouettes) for each gender. Adolescents are instructed to indicate the image that most closely resembles them (actual) and the one they desire to look like (ideal). The body silhouette item is scored with 1 for any discrepancy between the ideal and actual images and 0 for no difference. The extreme weight control behavior subscale consists of three items: dieting, exercising a lot, and purging/restricting behaviors, such as dieting, fasting, exercising, and using diet pills, diuretics, and laxatives. The items are scored as ‘yes’ = 1 and ‘no’ = 0, and the scores are summed to produce the total extreme weight control behavior subscale score. The maximum subscale score for negative weight attitudes is four, while the maximum subscale score for extreme weight control behaviors is three. The overall maximum M-KEDS score is seven. Higher scores indicate more disordered eating behaviors and negative weight attitudes. The M-KEDS has been validated through confirmatory factor analysis and internal consistency, as reported by Lopez et al. (Kuder-Richardson = 0.77) [31]. The M-KEDS is a self-report survey administered in a classroom setting, although it was not anonymous; however, confidentiality was assured. Students were instructed not to include identifying information on their survey packets, and surveys were coded using unique participant IDs.

#### 2.2.2. Demographic and Anthropometric Data

Participants provided their age, gender, and ethnicity through a self-report survey at baseline. Weights were measured using a digital scale, and participants were asked to wear light clothing and no shoes. Height was measured with a stadiometer, also without shoes. BMI was calculated from the measured height and weight. The body mass index z-score (zBMI), a standardized measure of body weight relative to height adjusted for age and gender, was calculated. BMI percentiles were determined based on gender-specific BMI-for-age percentiles for children aged 2 to 19 [32,33]. Due to the small sample size (*n* = 10) of the underweight group, these participants were excluded from this study. Both conceptual and statistical considerations guided this decision. Including them may have introduced heterogeneity that could obscure the primary relationships of interest in our models. Clinically, both overweight and obesity are associated with increased risk for body dissatisfaction and disordered eating behaviors, and they share similar psychosocial risk profiles in youth. Combining these categories is also consistent with prior literature examining weight-related attitudes and behaviors in adolescent populations. Given this study’s focus on weight dissatisfaction and disordered eating in relation to elevated body size, excluding this small subgroup (*n* = 10, approximately 1.4% of the sample) ensured a more conceptually and statistically coherent analysis. Additionally, merging these groups increased statistical power and ensured more stable estimates, particularly in subgroup analyses by gender.

### 2.3. Data Analysis

Data were analyzed using SPSS (Version 25, IBM, Armonk, NY, USA) [34], employing standard parametric and non-parametric methods. Chi-square tests compared weight status (Healthy weight versus overweight/obesity) and gender groups regarding the frequency of specific disordered eating behaviors measured by each item of the M-KEDS (‘yes’ versus ‘no’ responses). A Bonferroni correction was applied to the chi-square tests comparing individual M-KEDS item responses across gender and weight status (14 comparisons), adjusting the significance threshold to *p* < 0.0036 [35]. Cramér’s V effect sizes were computed to enhance interpretability for chi-square tests. Weight status and gender groups were assessed across the M-KEDS subscales and total scores using the Mann–Whitney U test.

A stepwise enter method logistic regression analysis was conducted to estimate a regression model that correctly predicts the probability of Hispanic adolescents’ disordered eating behaviors and body dissatisfaction behaviors based on the items of the M-KEDS. In total, three factors were entered into the analysis: weight status (healthy versus overweight/obese), gender, and the interaction between weight status and gender. Prior to conducting the analysis, chi-square and independent *t*-tests were used to examine the bivariate relationship between M-KEDS items and each factor. Furthermore, tolerance and VIF values were computed for all factors to examine the assumption of multicollinearity. Both tolerance and VIF values show that no multicollinearity problem exists among the factors. Finally, Hosmer and Lemeshow’s test of contingency table shows no cells had an expected value smaller than 1. Odds ratios (ORs) with 95% confidence intervals (CI) are presented for logistic regressions.

A stepwise multiple regression analysis was conducted to estimate a regression model that best predicts the M-KEDS subscale and total scores among Hispanic adolescents based on three factors: gender, weight status, and the interaction of weight status × gender. Prior to analysis, descriptive variables and graphs were examined to test assumptions of normality of residuals, linearity, homoscedasticity, and multicollinearity. Inspections of the histogram and the probability plots of residuals indicate that errors were normally distributed. Finally, an evaluation of the correlation matrix, VIF, and tolerance values ensures that no multicollinearity exists among the factors.

## 3. Results

### 3.1. Demographics

The study sample comprised 680 participants, of whom 53% were female. The participants were 9 to 15 years old (12.1 ± 0.7), and 100% identified as Hispanic. Within the sample, 50% (*n* = 346) had a healthy weight, 17% (*n* = 115) were overweight, and 32% (*n* = 219) had obesity. Among females, 45% had overweight or obesity (*n* = 162), while 53% of males had overweight or obesity (*n* = 172). Table 1 shows the participants’ demographics.

### 3.2. M-KEDS Items by Gender and Weight Status

Table 2 presents the percentage of adolescents who endorsed each item on the M-KEDS, stratified by gender and weight status. A total of 14 hypotheses were tested, and the Bonferroni-adjusted significance level was 0.05/14 = 0.003 [35]. Among females with overweight/obesity, the prevalence of negative weight attitudes was significantly higher compared to those with a healthy weight. Specifically, 90% of females with overweight/obesity reported wanting to lose weight, significantly more than healthy-weight females (χ^2^(1, *n* = 357) = 95.81, *p* < 0.001), with a large effect size (Cramer’s V = 0.52). They also reported feeling fat more frequently to others (χ^2^(1, *n* = 357) = 57.27, *p* < 0.001), with a medium effect size (Cramer’s V = 0.40). In addition, fear of weight gain was more common in this group (χ^2^(1, *n* = 357) = 26.90, *p* < 0.001), with a small effect size (Cramer’s V = 0.27). In addition to being more likely to report body dissatisfaction (χ^2^(1, *n* = 357) = 57.01, *p* < 0.001), with a medium effect size (Cramer’s V = 0.40), 60% of healthy weight females reported body dissatisfaction.

Extreme weight control behaviors among females with overweight/obesity were significantly different from those of females with a healthy weight. Specifically, 59% of females with overweight or obesity reported dieting to lose weight, compared to 19% of females with a healthy weight (χ^2^(1, *n* = 357) = 59.75, *p* < 0.001), with a medium effect size (Cramer’s V = 0.41). Overweight/obese females also reported excessive exercise more frequently for weight loss (χ^2^(1, *n* = 357) = 14.66, *p* < 0.001), with a small effect size (Cramer’s V = 0.20). In addition, purging or restrictive behaviors were also significantly more common among females with overweight/obesity (χ^2^(1, *n* = 357) = 9.60, *p* = 0.002), with a small effect size (Cramer’s V = 0.16).

Similar patterns were observed among males. The prevalence of negative weight attitudes was significantly higher in overweight/obesity compared to those with a healthy weight. Specifically, 86% of overweight/obese reported wanting to lose weight (χ^2^(1, *n* = 323) = 108.01, *p* < 0.001), with a large effect size (Cramer’s V = 0.58). They also reported feeling fat more frequently to others (χ^2^(1, *n* = 323) = 73.47, *p* < 0.001), with a large effect size (Cramer’s V = 0.48). In addition, fear of weight gain was more common in this group (χ^2^(1, *n* = 323) = 16.67, *p* < 0.001), with a small effect size (Cramer’s V = 0.23). In males, 90% of those with overweight/obesity reported body dissatisfaction compared to 52% of those with a healthy weight (χ^2^(1, *n* = 323) = 59.18, *p* < 0.001), with a medium effect size (Cramer’s V = 0.43).

Extreme weight control behaviors among males with overweight/obesity were significantly different from those among males with a healthy weight. Specifically, they were more likely to report dieting (χ^2^(1, *n* = 323) = 57.55, *p* < 0.001), with a medium effect size (Cramer’s V = 0.42). Males with overweight/obesity reported excessive exercise for weight loss at a rate of 64% (χ^2^(1, *n* = 323) = 50.46, *p* < 0.001), with a medium effect size (Cramer’s V = 0.40). In addition, purging or restrictive behaviors were also significantly more common among males with overweight/obesity (χ^2^(1, *n* = 357) = 8.47, *p* = 0.004), with a small effect size (Cramer’s V = 0.16).

#### M-KEDS Items by Gender and Weight Status Logistic Regression

The results of the stepwise logistic regression analysis using the enter method indicated that weight status consistently predicted all disordered eating and body dissatisfaction outcomes assessed by the M-KEDS. Table 3 presents the logistic regression odds ratios (95% CI) for M-KEDS items, categorized by predictors: weight status, gender, and the interaction between weight status and gender. Adolescents with overweight or obesity were significantly more likely to endorse these behaviors and attitudes. In the model predicting “wanting to lose weight now,” weight status emerged as the sole significant predictor (Wald(1) = 91.10, *p* < 0.001). For “feeling looked fat to others,” both weight status (Wald(1) = 63.45, *p* < 0.001) and gender (Wald(1) = 12.90, *p* < 0.001) were significant predictors. Similarly, in predicting “afraid to eat because of weight gain,” weight status (Wald(1) = 15.76, *p* < 0.001) and gender (Wald(1) = 4.80, *p* = 0.03) were both significant. “Body dissatisfaction” was significantly predicted by weight status alone (Wald(1) = 50.08, *p* < 0.001). For “dieted to lose weight,” weight status remained the only significant predictor (Wald(1) = 51.04, *p* < 0.001). In the model predicting “exercised a lot to lose weight,” both weight status (Wald(1) = 47.29, *p* < 0.001) and the interaction between weight status and gender (Wald(1) = 5.96, *p* = 0.02) were significant. Finally, for the combined behavior of “fasted, vomited, or used pills/diuretics/laxatives to lose weight,” weight status was again a significant predictor (Wald(1) = 8.00, *p* = 0.005). These findings underscore the heightened vulnerability of Hispanic adolescents with higher weight status to engage in disordered eating behaviors and to experience negative weight-related attitudes.

### 3.3. M-KEDS Subscale and Total Scores by Gender and Weight Status

Table 4 presents the mean scores and standard deviations for the two M-KEDS subscales—negative weight attitudes and extreme weight control behaviors—and the total M-KEDS score, stratified by gender and weight status. The results reveal significant differences in negative weight attitudes and extreme weight control Behaviors between adolescents with a healthy weight and those with overweight/obesity, for both females and males.

Across the total sample, the mean negative weight attitude score was 2.1 (SD = 1.4), and the mean extreme weight control behavior score was 0.9 (SD = 1.0). The overall M-KEDS total score averaged 3.0 (SD = 2.1), with noticeable variation by gender and weight category. Females’ component scores were significantly higher than males’ for the negative weight attitude subscale (Mann–Whitney U = 51,290.5, *p* < 0.05). The extreme weight control behavior subscale and the M-KEDS total score were not significantly different by gender.

Among females, adolescents with overweight or obesity had significantly higher scores than their healthy-weight peers on negative weight attitudes, extreme weight control behaviors, and the total M-KEDS score. Specifically, negative weight attitude scores were twice as high for those with overweight/obesity (M = 3.0, SD = 1.0) compared to those with a healthy weight (M = 1.5, SD = 1.3; *p* < 0.001). A similar pattern emerged for the extreme weight control behavior subscale (M = 1.2, SD = 1.0 vs. M = 0.5, SD = 0.8; *p* < 0.001) and the total M-KEDS score (M = 4.2, SD = 1.6 vs. M = 2.0, SD = 1.9; *p* < 0.001).

Among males, adolescents with overweight/obesity also scored significantly higher on all three measures. Negative weight attitudes were notably elevated (M = 2.7, SD = 1.0) compared to their healthy-weight counterparts (M = 1.1, SD = 1.1; *p* < 0.001). Extreme weight control behaviors were more prevalent as well (M = 1.4, SD = 0.9 vs. M = 0.5, SD = 0.8; *p* < 0.001), and this trend extended to the total M-KEDS score (M = 4.1, SD = 1.7 vs. M = 1.6, SD = 1.6; *p* < 0.001).

#### M-KEDS Subscale and Total Scores Multiple Regression Models

The results of the stepwise multiple regression analysis revealed that two of the three factors emerged as significant predictors for the M-KEDS subscale and total scores. Table 5 depicts the results for the model predicting negative weight attitudes. Weight status emerged as the strongest predictor, accounting for 31% of the variance in negative weight attitudes. Gender was also a significant predictor in this model, accounting for an additional 2% of the variance (R = 0.58, R^2^ = 0.33, Adj. R^2^ = 0.33, F(1, 677) = 19.84, *p* = 0.001). Table 6 depicts the results for the regression model predicting extreme weight control behaviors. In this model, only weight status emerged as a significant predictor, accounting for 17% of the variance in extreme weight control behaviors (R = 0.42, R^2^ = 0.17, Adj. R^2^ = 0.17, F(1, 678) = 140.95, *p* = 0.001). Table 7 shows the results for the multiple regression predicting the M-KEDS total score. In this model, weight status was the most significant predictor, accounting for 32% of the variance, and adding gender accounted for an additional 0.5% of the variance in the M-KEDS total score (R = 0.57, R^2^ = 0.32, Adj. R^2^ = 0.32, F(1, 677) = 5.60, *p* = 0.02).

## 4. Discussion

This study provides important insights into the prevalence of negative weight attitudes and extreme weight control behaviors among Hispanic adolescents, emphasizing notable differences by gender and weight status. Consistent with prior research, our findings revealed a high prevalence of body dissatisfaction and disordered eating behaviors in this population. Approximately 73% of adolescents endorsed body dissatisfaction, 60% reported wanting to lose weight, and 44% indicated feeling that they looked fat to others. These findings emphasize that negative weight attitudes are widespread among Hispanic adolescents, including those with a healthy weight.

The differences in negative weight attitude scores between healthy weight and overweight/obese adolescents reflect large effect sizes, indicating meaningful disparities in body dissatisfaction and weight-related concerns. Even moderate differences in extreme weight control behaviors may reflect important clinical risks in this population, particularly given the early age of onset. These findings highlight the urgency of early screening and intervention to prevent the escalation of disordered eating behaviors into clinically significant eating disorders.

As hypothesized, adolescents with overweight or obesity exhibited significantly higher scores for both negative weight attitudes and extreme weight control behaviors compared to their healthy-weight peers. This pattern was consistent across both males and females, reinforcing prior research that excess weight is associated with greater body dissatisfaction and engagement in unhealthy weight control behaviors [10,16,17,18]. Notably, females generally endorsed negative weight attitudes and extreme weight control behaviors at higher rates than males, except for exercising to lose weight, which was more common among males with overweight/obesity.

This finding likely reflects sociocultural and behavioral factors influencing male body image. While females are more likely to internalize thinness ideals, adolescent males may experience pressure to achieve a lean, muscular physique, which can lead to compensatory behaviors such as excessive exercise [16,24]. Cultural constructions of masculinity emphasize physical strength and athleticism, potentially normalizing appearance-driven physical activity in boys [36]. This gendered expression of body dissatisfaction may explain the higher rates of exercise to lose weight observed in our Hispanic male subgroup. Though socially reinforced, such behaviors may obscure underlying body image distress and risk for disordered eating.

Interaction analysis further revealed that gender significantly moderated the relationship between weight status and ‘Exercising to lose weight’. Females with overweight/obesity reported the highest levels of body dissatisfaction, while healthy-weight males reported the lowest. No significant interaction was observed for other items of the M-KEDS. These findings underscore the importance of examining not only main effects but also how gender and weight status intersect to shape risk profiles. These results extend previous findings suggesting that Hispanic males may experience body dissatisfaction at rates comparable to females but may express these concerns through different behaviors, such as excessive exercise [19,37]. This highlights the need for gender-sensitive screening tools and intervention strategies tailored to cultural and behavioral norms among Hispanic adolescents.

The high prevalence of unhealthy and extreme weight control behaviors, particularly among adolescents with overweight/obesity, is concerning. Approximately 13% of the sample reported engaging in extreme behaviors such as fasting, vomiting, and misuse of diet pills or laxatives, aligning with prior findings in similar populations [12,15]. These behaviors pose serious physical and psychological health risks and may precede the development of full-syndrome eating disorders [5,6].

Moreover, the presence of disordered eating behaviors among adolescents with a healthy weight indicates that weight status alone is not protective. Therefore, prevention efforts must target all adolescents, regardless of weight status, and promote body positivity and healthy behaviors across the weight spectrum.

Cultural norms and family dynamics likely mediate gender differences in weight-related attitudes and behaviors among Hispanic youth. In many Hispanic communities, traditional gender roles pressure girls toward thinness and boys toward strength. Weight-related family comments, particularly toward daughters, have been associated with increased risk for body dissatisfaction and disordered eating [18]. For boys, culturally sanctioned norms around muscularity may lead to greater acceptance of excessive exercise, even when rooted in body dissatisfaction [22,23,24].

In addition to sociocultural and behavioral explanations, emerging research highlights the role of complex emotional processes—particularly guilt—as important psychological drivers of disordered eating behaviors in adolescents. While guilt was not directly measured in the current study, it may serve as a key emotional antecedent to body dissatisfaction and maladaptive weight control behaviors. Guilt related to eating, appearance, or perceived lack of self-control has been shown to exacerbate negative self-evaluation. It may reinforce extreme behaviors such as purging or excessive exercise as a means of emotional regulation. Raffone et al. (2025) found that guilt was significantly associated with disordered eating symptomatology in adults, underscoring its relevance as a transdiagnostic factor across different eating disorder presentations [38]. Including this emotional dimension in future research may enrich our understanding of the psychological mechanisms underlying negative weight attitudes and extreme weight control behaviors in Hispanic youth, and could inform more targeted interventions that address not only body image but also internalized emotional distress.

Despite the high prevalence of disordered eating and body dissatisfaction in this population, many Hispanic adolescents face barriers to care [16,39]. Cultural stigma around mental health, lack of access to culturally competent services, language barriers, and financial constraints may prevent adolescents from seeking help [9,22]. Additionally, boys may be less likely to disclose symptoms due to the misconception that eating disorders primarily affect females [24]. These intersecting barriers point to the need for school-based screening, family engagement, and culturally tailored interventions.

From a clinical and public health perspective, the findings support the implementation of early screening programs in school settings, particularly in Hispanic-serving districts. Training educators and healthcare providers to recognize culturally and gender-specific symptoms is essential. Prevention efforts should address sociocultural ideals, support positive body image, and promote healthy behaviors while dismantling stigma around disordered eating.

This study has several strengths, including a large, ethnically homogenous sample, direct anthropometric measurements, and use of the M-KEDS, a culturally validated tool for Hispanic adolescents. These design features improve upon earlier studies that often relied on heterogeneous samples or non-validated tools, thereby strengthening the cultural validity and clinical utility of the findings [9,14].

However, limitations must be acknowledged. This study lacked clinician-administered assessments; however, the self-reported M-KEDS remains well-suited for the population studied. The cross-sectional design prevents causal inference, and the single-site convenience sampling limits generalizability. Although the sample was predominantly Mexican-American, the findings may not be generalizable to other Hispanic subgroups. Additionally, the dichotomous M-KEDS response format may underestimate behavior frequency and clinical severity. While subscale and total scores help capture cumulative risk, future research should incorporate frequency-based measures to assess severity and clinical thresholds better. Moreover, while M-KEDS is validated for Hispanic ethnicity, it is not tailored to adolescents with overweight or obesity, highlighting the need for screening tools sensitive to both cultural and weight-related factors.

## 5. Conclusions

These findings have important implications for future research, clinical practice, and public health strategies targeting Hispanic adolescents. This study reinforces and expands existing evidence that disordered eating behaviors and negative weight attitudes are highly prevalent in this population, especially among youth with overweight or obesity. Given this elevated risk, there is a critical need for culturally responsive prevention, early identification, and intervention programs.

However, resource constraints in low-income or under-resourced school settings may pose challenges to routine screening. To address these barriers, feasible and culturally appropriate strategies—such as brief, validated tools like the M-KEDS—can be implemented in school and community settings. Additionally, low-cost, family-focused workshops offer promising avenues for increasing awareness and identifying adolescents at risk.

Schools remain a vital setting for prevention and early detection, particularly in predominantly Hispanic communities. Because disordered eating behaviors often begin during early adolescence and may go unrecognized, training school personnel, including counselors, nurses, and PE teachers, to identify culture- and gender-specific warning signs is essential [40]. Routine screening using culturally validated tools can help detect problems before they escalate, while family-based education can reinforce healthy behaviors and improve help-seeking [41].

Importantly, Hispanic adolescents, especially males, are less likely to be screened or seek help for disordered eating symptoms. This highlights the need for policies that prioritize early detection, reduce stigma, and ensure access to culturally competent care [40].

Future research should focus on the development and evaluation of school-based and family-centered interventions that are both culturally tailored and gender sensitive [18]. Such interventions should address body image concerns and disordered eating behaviors simultaneously, potentially serving as protective factors against the dual burden of adolescent eating disorders and adult obesity. A comprehensive and integrated approach is essential for improving long-term health outcomes and reducing disparities among Hispanic youth.

## Figures and Tables

**Table 1:** Demographics of female and male Hispanic adolescents (*n* = 690).

	Females (*n* = 357) (mean ± SD)	Males (*n* = 323) (mean ± SD)
Age	12.0 ± 0.6	12.1 ± 0.7
BMI	22.4 ± 5.5	22.7 ± 5.6
zBMI	0.81 ± 1.1	0.97 ± 1.1
Healthy weight	55%	47%
Overweight/obesity	45%	53%

**Table 2:** Percentage of adolescents endorsing negative weight attitudes and extreme weight control behaviors by gender and weight status.

	Total Sample	Females			Males		
Items	Percent Endorsed (*n* = 680)	Healthy Weight (*n* = 195), %	Overweight/Obesity (*n* = 162), %	X^2^	Healthy Weight (*n* = 151), %	Overweight/Obesity (*n* = 172), %	X^2^
**Negative Weight Attitudes**							
Wanted to lose weight now	61	39	90	95.8 *	29	86	108.0 *
Felt looked fat to others	44	31	71	57.3 *	14	61	74.5 *
Afraid to eat because of weight gain	29	23	49	26.9 *	13	33	16.7 *
Body Dissatisfaction	74	59	94	57.0 *	52	90	59.2 *
**Extreme Weight Control Behaviors**							
Dieted to lose weight	37	19	59	59.7 *	15	55	57.6 *
Exercised a lot to lose weight	39	24	43	14.7 *	25	64	50.5 *
Fasted to lose weight, vomited to lose weight, used diet pills, used diuretics, or used laxatives	13	7	18	9.6 *	8	19	8.5

* *p* ≤ 0.003, chi-square test; df = 1.

**Table 3:** Logistic regression odds ratios (95% CI) for M-KEDS items by predictors: weight status, gender, and the interaction of weight status and gender.

Dependent Variables	Weight Status OR [95% CI]	Gender OR [95% CI]	Weight × Gender OR [95% CI]
Want to lose weight now	15.00 [8.60–26.15] **	1.55 [0.99–2.45]	0.89 [0.40–1.99]
Feel fat to others	9.47 [5.45–16.46] **	2.75 [1.58–4.78] **	0.58 [0.28–1.19]
Afraid to eat because of weight gain	3.16 [1.79–5.58] **	1.91 [1.07–3.40] *	1.03 [0.50–2.14]
Body dissatisfaction	8.53 [4.71–15.45] **	1.35 [0.88–2.06]	1.23 [0.50–3.10]
Dieted to lose weight	7.23 [4.20–12.45] **	1.37 [0.74–2.44]	0.84 [0.41–1.72]
Exercise to lose weight	5.47 [3.37–8.87] **	0.98 [0.60–1.61]	0.44 [0.23–0.85] *
Extreme weight control behaviors	2.75 [1.36–5.55] *	0.90 [0.40–2.00]	1.03 [0.39–2.72]

Reference category for gender is male; * *p* < 0.01, ** *p* < 0.001.

**Table 4:** Mean total and subscale scores on the M-KEDS.

	Total Sample	Females		Males	
	Mean± SD	Mean ± SD		Mean ± SD	
M-KEDS		Healthy	Overweight/Obese	Healthy	Overweight/Obese
Negative Weight Attitudes	2.1 ± 1.4	1.5 ± 1.3	3.0 ± 1.0 *	1.1 ± 1.1	2.7 ± 1.0 *
Extreme Weight Control Behaviors	0.9 ± 1.0	0.5 ± 0.8	1.2 ± 1.0 *	0.5 ± 0.8	1.4 ± 0.9 *
Total score	3.0 ± 2.1	2.0 ± 1.9	4.2 ± 1.6 *	1.6 ± 1.6	4.1 ± 1.7 *

* *p* < 0.001.

**Table 5:** Multiple linear regression predicting negative weight status.

Predictor	B	SE B	β	t	*p*
Intercept	1.11	0.08	–	14.20	0.001
Weight status	1.56	0.09	0.57	18.03	0.001
Gender	0.39	0.09	0.14	4.45	0.001

Note. B = unstandardized coefficient; SE B = standard error of the coefficient; β = standardized beta coefficient. Gender is coded as 0 = male and 1 = female.

**Table 6:** Stepwise multiple linear regression predicting extreme weight control behaviors.

Predictor	B	SE B	β	t	*p*
Intercept	0.49	0.05	–	10.28	0.001
Weight status	0.81	0.07	0.42	11.87	0.001

Note. B = unstandardized coefficient; SE B = standard error of the coefficient; β = standardized beta coefficient. Gender is coded as 0 = male and 1 = female.

**Table 7:** Stepwise multiple linear regression predicting M-KEDS total score.

Predictor	B	SE B	β	t	*p*
Intercept	1.64	0.12	–	13.90	0.001
Weight status	2.40	0.13	0.57	18.00	0.001
Gender	0.31	0.13	0.08	2.40	0.02

Note. B = unstandardized coefficient; SE B = standard error of the coefficient; β = standardized beta coefficient. Gender is coded as 0 = male and 1 = female.

## Data Availability

Upon request, data may be made available after a data use agreement.

## Abbreviations

The following abbreviations are used in this manuscript:

M-KEDS	Modified Kids Eating Disorder Survey
